# Regression analysis with categorized regression calibrated exposure: some interesting findings

**DOI:** 10.1186/1742-7622-3-6

**Published:** 2006-07-04

**Authors:** Ingvild Dalen, John P Buonaccorsi, Petter Laake, Anette Hjartåker, Magne Thoresen

**Affiliations:** 1 lnstitute of Basic Medical Sciences, Department of Biostatistics, University of Oslo, P.O. Box 1122, Blindern, 0317 Oslo, Norway; 2 Department of Mathematics and Statistics, University of Massachusetts, 710 North Pleasant Street, Amherst, MA 01003-9305, USA; 3 Institute of Community Medicine, Faculty of Medicine, University of Tromsø, 9037 Tromsø, Norway

## Abstract

**Background:**

Regression calibration as a method for handling measurement error is becoming increasingly well-known and used in epidemiologic research. However, the standard version of the method is not appropriate for exposure analyzed on a categorical (e.g. quintile) scale, an approach commonly used in epidemiologic studies. A tempting solution could then be to use the predicted continuous exposure obtained through the regression calibration method and treat it as an approximation to the true exposure, that is, include the categorized calibrated exposure in the main regression analysis.

**Methods:**

We use semi-analytical calculations and simulations to evaluate the performance of the proposed approach compared to the naive approach of not correcting for measurement error, in situations where analyses are performed on quintile scale and when incorporating the original scale into the categorical variables, respectively. We also present analyses of real data, containing measures of folate intake and depression, from the Norwegian Women and Cancer study (NOWAC).

**Results:**

In cases where extra information is available through replicated measurements and not validation data, regression calibration does not maintain important qualities of the true exposure distribution, thus estimates of variance and percentiles can be severely biased. We show that the outlined approach maintains much, in some cases all, of the misclassification found in the observed exposure. For that reason, regression analysis with the corrected variable included on a categorical scale is still biased. In some cases the corrected estimates are analytically equal to those obtained by the naive approach. Regression calibration is however vastly superior to the naive method when applying the medians of each category in the analysis.

**Conclusion:**

Regression calibration in its most well-known form is not appropriate for measurement error correction when the exposure is analyzed on a percentile scale. Relating back to the original scale of the exposure solves the problem. The conclusion regards all regression models.

## Introduction

Measurement error is recognized as a common problem in epidemiological studies. Many interesting variables are registered with a relatively large degree of uncertainty, often due to low-price and simple measurement methods. The errors could be either random (e.g. due to biological fluctations about a mean), systematic (e.g. due to varying calibrations of measurement instruments), or both, which is most often the case. It is well known that measurement error in predictors biases effect estimates in regression modelling. For this reason, measurement error has been the subject of extensive research over the recent decades, and several methods have been proposed for handling the problem. In linear models the standard reference is [1], while Carroll *et al. *[2] provide an excellent overview of methods applying to non-linear models.

One of the methods for dealing with measurement error that has gained popularity is the so-called *regression calibration *method; see for example Chapter 3 of [2]. This is most likely due to its intuitive nature, relative ease of use and general applicability. It has also been shown to have good properties in many situations. Regression calibration was introduced to the epidemiologic community by Rosner *et al. *[3,4]. In another formulation of the same method [5], the idea is to predict the unobservable error-prone variable by means of regression, and then to include this predicted variable in the main analysis. The approach involves efforts to somehow relate the observed variable to the underlying "true" variable, either through a sub validation study where the true value is observed directly for some of the individuals, through repeated measurements for some or all of the individuals, or by use of so-called instrumental variables that supply information about the true values relative to the measured values. It is also possible to apply information from external sources. Software for performing regression calibration is available in STATA [6] and in SAS [7,8].

The most well-known version of regression calibration is the one developed for continuous explanatory variables. However, in epidemiological studies it is also common to categorize the exposure variables according to rank such as quintiles; a selection of newer examples of studies using this approach is [9-13]. Usually an analysis comparing each quintile group to the lowest (reference) group is supplemented with a test for trend for the quintile numbers. Another trend estimator applies the median values of the quintile groups [14,15]. The reason for categorizing the exposure could be to obtain analyses that require less stringent assumptions and that are more robust to outlying values [16]. Now that regression calibration is becoming more standard in the epidemiologic community, one can easily imagine a situation where this method is applied to a continuous variable, which is subsequently categorized before it is incorporated in the main (regression) analysis. The researcher might then feel confident that he or she has taken the necessary precautions with regard to measurement error.

We study the performance of this approach under 3 different modelling schemes, all applying the same categorization according to quintiles: regression on (A) dummy variables, (B) quintile numbers, and (C) median value within quintile groups, thereby obtaining what one may call an enhanced trend estimator. The corresponding results from analyses with the continuous exposure are included for comparison. Linear regression is used as the framework for our demonstration, but, as will be shown, the results are valid for other regression models as well.

We find that for analysis with dummy variables and for simple trend analysis, in most cases the corrected effect estimates are approximately equal to the ones obtained without making the correction. In some cases they are identical. We argue that categorizing the corrected exposure still retains misclassification similar to the misclassification obtained using the observed exposure. This misclassification induces bias in the effect estimates. When introducing the median value of each category to the analysis, the correction method regains some of its usual advantage over the naive approach. The reason for this will become clear.

We start off defining the models used, and then present analytical and semi-analytical arguments and results for the various settings defined above. The results are illustrated by simulated examples and also by a real-life example, where we have examined the relationship between folate intake and risk of depression in a prospective cohort study of Norwegian women, the Norwegian Women and Cancer study (NOWAC).

## Methods

In the following we will assume that an exposure variable *X *is measured with error and in effect is unobservable. The true exposure *X *is instead observed through a measured value *W*, and we assume an additive error model such that *W *= *X *+ *U*, where *U *is the measurement error, with expected value *E *(*U*) = 0. We also observe a response or disease variable *Y *and sometimes a covariate *Z*, both measured without error. Importantly, we assume that the measurement error is non-differential, i.e., *F *(*W*|*X*, *Y*) = *F *(*W|X*). This implies that *W *contributes no new information about *Y *apart from what is already in *X*.

The idea of regression calibration [3-5,17-19] is to predict the unobservable variable *X *by means of regression, and then to include this predicted variable in the main analysis. As such, it is applicable to any regression modelling setting. Extra information needs to be supplied in order to relate the true variable to the observed error-prone variable. We assume we have replicated measures of the exposure. That is, we assume that for individual *i *there exist *k*_*i *_replicate measurements of *X*_*i*_, given by *W*_*ij *_= *X*_*i *_+ *U*_*ij*_; *j *= 1,..., *k*_*i*_, *i *= 1, ..., *n*. Their mean is _*i*_. The replicates are assumed to be uncorrelated given *X*. Following [2], in cases with replicated data, the best linear predictor of *X *given  and *Z*, is given by



where *μ*_*X*_, *μ*_*W *_and *μ*_*Z *_denote the expected values of *X*, *W *and *Z*, respectively; ,  and  are the variances of *X*, *U *and *Z*; and finally *σ*_*XZ *_denotes the covariance between *X *and *Z*. Since *E *(*U*) = 0, *μ*_*X *_= *μ*_*W*_. Equation (1) defines the *RC *predictor for the error-prone exposure *X*. The parameters in (1) must be estimated from the data, e.g. as described in [2], pages 47–48, or see [6] for a detailed procedure in STATA.

The true exposure *X *and the covariate *Z *are assumed to be associated with the response variable *Y *in a regression model. In the case of a linear regression model, the relation between the continuous *X *and *Z *and the continuous *Y *is given by

*E *(*Y*) = *β*_0 _+ *β*_1 _*X *+ *β*_2_*Z*.     (2)

However, as mentioned, we are interested in estimating the effects of exposure categorized according to quintiles. We define three modelling schemes as follows: In model A we apply dummy variables to see separately the effects of the different quintile groups compared to the lowest (reference) group:

*E *(*Y*) = *α*_0 _+ *α*_1_*I*_1 _+ *α*_2_*I*_2 _+ *α*_3_*I*_3 _+ *α*_4_*I*_4 _+ *α*_5_*Z*,     (3)

where *I*_*r *_is 1 if *x *∈  and 0 otherwise. *F*_*X *_denotes the cumulative distribution of *X*, hence  is the *r*th quintile point in the distribution of *X*. When evaluating the performance for this method, we mainly look at *α*_4_, which is the difference in mean response between the extreme quintile groups for the exposure. The covariate *Z *is still analyzed on the continuous scale.

Using model B we will obtain a simple trend estimator for the exposure, which is often supplemented to the effect estimates from model A. We write

*E *(*Y*) = *γ*_0 _+ *γ*_1_*X*_*c *_+ *γ*_2_*Z*,     (4)

where *X*_*c *_is a number from 0 to 4, according to which quintile group *X *falls into. Hence, if *x *∈  then *x*_*c *_= *r*.

The trend estimator in model C is an enhancement from the one in model B, in that it retains some of the information from the original continuous measurements, but still deals with extreme values and skewed exposure distributions. The model is defined by

*E *(*Y*) = *ψ*_0 _+ *ψ*_1_*X*_*med *_+ *ψ*_2_*Z*,     (5)

where *X*_*med *_are the median values of the individuals falling into the various categories. Hence, if *x *∈ , then *x*_*med *_is assigned the median value of all the individuals in the *r*th quintile group.

When comparing the effect estimates obtained from fitting a regression model involving the categorized *RC *predictor as the exposure to those obtained using a naive predictor () and to the true effect estimates (obtained from *X*), we categorize the two former according to quintiles in their respective distributions. Hence, the cut-points for the naive predictor and the *RC *predictor will in general not be the same as the ones for the true exposure. Neither will the median values.

If the response variable *Y *is instead dichotomuous, e.g. representing a disease variable where the value 1 is assigned to diseased individuals and 0 is assigned to healthy ones, we must replace *E *(*Y*) with the logit transform *log *[*E *(*Y*)/(1 - *E *(*Y*)]. Similar transforms apply to other regression models.

With respect to standard errors for the *RC *corrected estimates, these will be underestimated by ordinary methods as they do not take into account the variance in the estimation of *X*. Since the computation of explicit formulas for the standard error is quite tedious [5], standard errors are typically obtained through bootstrapping [2,20].

## Results

### Analytical results

In a situation without additional covariates, Equation (1) simplifies considerably. We can write



where the factor  is a modified version of the *reliability ratio*, usually defined as . In the following we look first to the situation where all individuals are measured the same number of times, in which case we obtain analytical results for all models A-C. When we allow the number of replicates to vary, we must rely on semi-analytical methods to make inferences.

#### Constant number of replicates

When all individuals are measured an equal number of times (*k*_*i *_= *k*), we find that the *RC *predictor  given in Equation (6) is simply a linear transformation of the naive predictor . This transformation represents in essence a weighting between the estimated sample mean and the individual means for each data point. Given a certain error (); when *k *is large and  thus relatively close to 1, relatively large confidence is put on the individual means and little correction is made. On the other hand, when *k *is small, all data points are adjusted closer to the sample mean. In both cases the adjustment is the same for all subjects, resulting in a distribution that is squeezed towards the estimated sample mean, as compared to the distribution of measured values.

The variance of  is given by



which is greater than *Var *(*X*) whenever *σ*_*U*_* > *0, that is, when there is measurement error. Notice also that when *k *→ ∞, *Var *() → *Var *(*X*); that is, if we were to have infinitely many replications, we would be able to estimate *Var *(*X*) without bias, using the observed values.

Furthermore, the variance of  is given by

*Var *() = *Var *(*λ'*) = *λ'*^2^*Var *() = *λ'Var *(*X*).

Thus, generally, the variance of  underestimates the variance of the exposure, in contrast to the variance of , which overestimates it.

Relating this adjusted continuous exposure to a response in a regression analysis results in larger effect estimates as compared to the ones obtained using the measured exposure. For example, in linear regression the effect is decided by the ratio of the covariance of exposure and response to the variance of the response (*σ*_*XY*_/), and even though the covariance between the corrected exposure and the response () underestimates *σ*_*XY *_due to measurement error, this is counteracted by the decreased variance of , resulting in unbiased effect estimates. Using the observed exposure, we get a so-called attenuated effect estimate, which is underestimating the true effect by a factor *λ' *[2].

However, when  and  are categorized according to percentiles in their respective distributions, we have a new problem. Since  is merely a linear transformation of , naturally any percentile point  in the distribution of  is given by the same linear transformation of the corresponding percentile point  in the distribution of . Hence, categorized according to quintile groups, _*c *_and _*c *_are the same. Consequently, effect estimates of dummy variables and quintile numbers in models A and B will be equal for the naive and the *RC *approach. This is valid for all types of response variables.

When it comes to using the medians of each quintile as explanatory variable, as proposed in model C, regression calibration regains some of its usual superiority over naive analyses. As explained, *RC *involves a squeezing of values towards the mean, so the distances between the medians in the distribution of corrected exposure will be smaller than in the naive distribution. Hence, corrected effect estimates will be larger than naive estimates.

Since the spread in the distribution of  underestimates the spread in the true exposure distribution, naturally the distances between median points in groups are also underestimated. However, as with the continuous case, this is counteracted by decreased covariance with the response.

We illustrate this using linear regression. If *X *~ *N *(0, ) and *U *~ *N *(0, ), and we have *k *replicates, then  ~ *N *(0, /*λ'*) and  ~ *N *(0, *λ'*). Hence, for any percentile point *q *we have that  and . Hence, for variables consisting of median points in quintile groups we have that *Var *(_*med*_) = *Var *(*X*_*med*_)/*λ' *and *Var *(_*med*_) = *λ' Var *(*X*_*med*_).

Regarding the covariances, we have that given that the error in the exposure is independent of the response *Y *(nondifferential measurement error), *Cov *(, *Y*) = *Cov *(*X*, *Y*). Thus, the covariance between the response and the variable given by medians in quintile groups of the naive exposure is

*Cov *(_*med*_, *Y*) = *Cov *(*X*_*med*_, *Y*).

Furthermore, using that the correlation between _*med *_and *Y *equals the correlation between _*med *_and *Y*, we find that the covariance between _*med *_and *Y *is

*Cov *(_*med*_,*Y*)= *λ' Cov *(*X*_*med*_, *Y*),

Hence, since in this case *Cov *(_*med*_, *Y*)/*Var *(_*med*_) * = Cov *(*X*_*med*_, *Y*)/*Var *(*X*_*med*_), the regression calibrated effect estimate is asymptotically correct. The naive estimates are on the other hand attenuated by the same factor Λ as when analyzing the exposure on continuous scale.

#### Varying numbers of replicates

When the number of replicates varies between individuals, we have in addition a kind of *confusion *effect, in that some data points are adjusted to a larger extent than others. However, the main effect of the transformation is the mentioned adjustment towards the sample mean. At least, we propose that classification of the corrected predictor  according to quintiles leads to much the same classification pattern as classification of the naive predictor .

To uphold the previous proposal, Table 1 displays the results of a simulated example, where for various replication patterns we have obtained the percentages of corresponding classifications between *X*_*c *_and _*c*_, *X*_*c *_and _*c*_, and _*c *_and _*c*_, respectively. We used *X *~ *N *(0, 1) and *U *~ *N *(0,1), and the number of replications was either 5 or 1. The total number of individuals was *n *= 100000, divided in various ways between the two replication groups. As can be seen from the table, most of the individuals were classified equally for the naive and the regression calibrated predictors. The exact figures vary depending on the replication pattern and which group the individuals belong to, the replicated or the nonreplicated ones, and finally which of these groups is larger and thus dominant in deciding the spread in the distribution of .

**Table 1:** Misclassification. Percentages of equal classifications between *X*_*c *_and _*c*_, *X*_*c *_and _*c *_and _*c *_and _*c *_for various replication patterns, where *X*_*c *_is the categorized true exposure, _*c *_is the categorized mean measured exposure, and _*c *_is the categorized *RC *corrected exposure, all of them categorized according to quintiles in the individual distributions.

*Pattern*	*applying to*	*x*_*c *_= _*c *_(%)	*x*_*c *_= _*c *_(%)	_*c *_= _*c *_(%)
1	total sample	44.3	44.1	89.1
	20% with 5 reps	58.3	58.1	72.7
	80% with 1 rep	40.8	40.6	93.2
				
2	total sample	55.6	55.2	89.1
	80% with 5 reps	59.4	59.3	93.2
	20% with 1 rep	40.2	38.8	72.7
				
3	total sample	50.3	49.7	83.0
	50% with 5 reps	59.5	58.9	83.0
	50% with 1 rep	41.0	40.5	83.0

At the same time, we see that the percentages of cases that are correctly classified (that is, in accordance with the classification of the true *X*), are very similar for the naive and the corrected predictors. Hence, categorizing using the corrected exposure still retains misclassification, and the magnitude of this is very similar to the misclassification obtained with the naive approach. Hence, the estimates relating to categorical exposure in models A and B, will be very similar for the naive and the *RC *approach. However, in model C, regression calibration still benefits from the mentioned squeezing of values towards the mean.

### Illustration with simulated data

We simulated a variety of situations to obtain numerical results regarding the biases of the naive and the corrected effect estimates. These simulations were conducted using the software program R version 2.2.1 [21], in which the base integrated routine for general linear models was applied to generated datasets of size *n *= 100000.

The true exposure *X *and the response *Y *were both generated from standard normal distributions. The error *U *was normally distributed with mean zero and variance  decided by various fixed levels of the reliability ratio . The covariate *Z *was omitted.

We studied cases where the correlation *ρ*_*XY *_between the response and the true continuous exposure, and hence the effect *β*_1_, was either 0.7 or 0.2, see Equation (2). These cases correspond to true mean differences *α*_4 _of 1.96 and 0.56 between the extreme quintiles in model A (Equation (3)), naive trends *γ*_1 _of 0.47 and 0.13 (model B, Equation (4)), and effects *ψ*_1 _of 0.76 and 0.22 using medians in groups as explanatory variables (model C, see Equation (5)).

Results were produced for three levels of the reliability ratio *λ*: 0.2 (which corresponds to a rather large measurement error), 0.5, and 0.8 (modest measurement error situation). Standard errors for the corrected effect estimates are obtained via resampling pairs bootstrapping with 200 bootstrap samples [20].

Two replication patterns were studied. First, we simulated situations where all individuals were measured twice, that is *k*_*i *_= *k *= 2. Next, we looked at situations in which a random 20% subset of the individuals are measured 5 times, while the rest only had 1 measurement (replication pattern 1 from Table 1). All the results are given in Table 2.

**Table 2:** Results from simulations without covariates. Naive and regression calibrated effect estimates in linear regression with error-prone exposure *X*, analysing (A) dummy variables, comparing 5th vs. 1st quintile, (B) quintile numbers, and (C) median values within quintile groups. Results from analysis with continuous exposure is included for comparison. We have *X *and *Y *~ *N *(0,1) and the error *U *~ *N *(0, ), where  is chosen such that the reliability ratio *λ *is either 0.8, 0.5 or 0.2. The results are obtained via simulation, where the correlation *ρ*_*XY *_between continuous *X *and *Y *is set to either 0.7 or 0.2. The true effects are indicated. For the cases marked '*k *constant', each individual is measured twice. For the cases marked '*k *not constant', the replication pattern is 5 measurements on a random 20% subset of individuals and 1 measurement on the rest. Standard errors for the corrected cases are bootstrapped.

				*k *constant		*k *not constant	
*ρ*_*XY*_	model	true *coef*	*λ*	naive *coef *(*SE*)	*RC coef *(*SE*)	naive *coef *(*SE*)	*RC coef *(*SE*)
			0.8	0.62 (0.003)	0.70 (0.002)	0.58 (0.003)	0.70 (0.003)
	cont	0.70	0.5	0.47 (0.003)	0.70 (0.004)	0.38 (0.002)	0.70 (0.005)
			0.2	0.23 (0.002)	0.69 (0.010)	0.16 (0.002)	0.69 (0.011)
							
			0.8	1.85 (0.010)	1.85 (0.008)	1.77 (0.010)	1.77 (0.008)
	A	1.96	0.5	1.61 (0.010)	1.61 (0.009)	1.45 (0.010)	1.47 (0.009)
			0.2	1.14 (0.010)	1.14 (0.008)	0.93 (0.010)	1.00 (0.010)
0.7							
			0.8	0.44 (0.002)	0.44 (0.002)	0.42 (0.002)	0.42 (0.002)
	B	0.47	0.5	0.38 (0.002)	0.38 (0.002)	0.35 (0.002)	0.35 (0.002)
			0.2	0.27 (0.002)	0.27 (0.002)	0.23 (0.002)	0.24 (0.002)
							
			0.8	0.68 (0.003)	0.76 (0.003)	0.62 (0.003)	0.76 (0.003)
	C	0.76	0.5	0.51 (0.003)	0.76 (0.005)	0.42 (0.003)	0.76 (0.006)
			0.2	0.25 (0.002)	0.75 (0.011)	0.18 (0.002)	0.77 (0.013)
							
			0.8	0.18 (0.003)	0.20 (0.003)	0.17 (0.003)	0.21 (0.003)
	cont	0.20	0.5	0.13 (0.003)	0.20 (0.004)	0.11 (0.002)	0.20 (0.004)
			0.2	0.07 (0.002)	0.20 (0.006)	0.04 (0.002)	0.19 (0.006)
							
			0.8	0.52 (0.010)	0.52 (0.010)	0.53 (0.010)	0.52 (0.010)
	A	0.56	0.5	0.45 (0.010)	0.45 (0.010)	0.41 (0.010)	0.42 (0.010)
			0.2	0.32 (0.010)	0.32 (0.010)	0.25 (0.010)	0.27 (0.009)
0.2							
			0.8	0.13 (0.002)	0.13 (0.002)	0.12 (0.002)	0.12 (0.002)
	B	0.13	0.5	0.11 (0.002)	0.11 (0.002)	0.10 (0.002)	0.10 (0.002)
			0.2	0.08 (0.002)	0.08 (0.002)	0.06 (0.002)	0.06 (0.002)
							

			0.8	0.19 (0.003)	0.22 (0.004)	0.18 (0.003)	0.22 (0.004)
	C	0.22	0.5	0.14 (0.003)	0.21 (0.005)	0.12 (0.003)	0.22 (0.005)
			0.2	0.07 (0.002)	0.22 (0.007)	0.05 (0.002)	0.21 (0.007)

We see that in situations with a constant number of replicates, regression calibration estimates are equal to the ones obtained from the naive approach, unless the original scale of measurement is somehow incorporated. None of the methods performed very poorly as long as the measurement error was not too large, however the effects were attenuated by a factor of almost 0.6 in both models A and B in the most severe measurement error situation studied (*λ *= 0.2). When *λ *= 0.5, the attenuation factor for these models was just above 0.8. Hence, the effect estimates differ considerably from the true effects in many cases. Moreover, a decrease in the reliability ratio is associated with increased bias, as was to be expected.

Using the median values in model C, we see that the regression calibration approach gives unbiased effect estimates. This is in contrast to the naive approach, which in the most severe cases (*λ *= 0.2) indicates effects that are about 1/3 of the true effects.

When the number of replicates varies, we see again that the regression calibration fails to improve significantly the effect estimates relative to the naive approach, except for with model C. In these results we see some small, though not substantial, differences between the two approaches for models A and B, due to the confusion effect mentioned previously. We also see that, in contrast to what could be expected from Table 1, it is the regression calibrated estimates that are slightly better off. Although the naive approach gives a higher percentage of correctly classified cases, the mean squared distance between the true and the observed category is actually larger than for the *RC *approach (1.23 vs. 1.20), explaining this apparent inconsistency. Notice also that the results are generally worse with this replication pattern than when all individuals were measured twice.

#### Including a covariate

Regression calibration uses the information of covariates in the correction procedure, see Equation (1). Thus, including a variable correlated to *X *in the analysis will probably give *RC *an advantage relative to the naive approach, especially when the correlation is strong.

We study the performance of regression calibration in the presence of a standard normal covariate *Z*, measured without error. The effect of *Z *is set to be equal to the effect of *X*, and the correlation *ρ*_*XZ *_between *X *and *Z *is either 0.2 or 0.7. Otherwise the situations are the same as in the previous examples, although we confine to situations with constant number of replicates (*k *= 2). The results are shown in Tables 3 (*ρ*_*XZ *_= 0.2) and 4 (*ρ*_*XZ *_= 0.7).

**Table 3:** Results from simulations including a covariate *Z*, weakly correlated to *X*. Naive and regression calibrated effect estimates in linear regression with error-prone exposure *X *and a covariate *Z *weakly correlated to *X *(*ρ*_*XZ *_= 0.2), analysing (A) dummy variables, comparing 5th vs. 1st quintile, (B) quintile numbers, and (C) median values within quintile groups. Results from analysis with continuous exposure is included for comparison. We have *X*, *Z *and *Y *~ *N *(0,1) and the error *U *~ *N *(0, ), where  is chosen such that the reliability ratio λ is either 0.8, 0.5 or 0.2. The results are obtained via simulation, where the correlations *ρ*_*XY *_= *ρ*_*ZY *_are set to either 0.7 or 0.2. The true effects of *X *and *Z *are indicated for the various models. All individuals are measured twice. Standard errors for the corrected cases are bootstrapped.

*ρ*_*XY *_= *ρ*_*ZY *_	model	true *coef *(*X*)	*λ*	naive *coef *(*X*) (*SE*)	*RC coef *(*X*) (*SE*)	true *coef *(*Z*)	naive *coef *(*Z*) (*SE*)	*RC coef *(*Z*) (*SE*)
			0.8	0.52 (0.001)	0.58 (0.002)		0.60 (0.002)	0.58 (0.001)
	cont	0.58	0.5	0.38 (0.001)	0.58 (0.003)	058	0.62 (0.002)	0.58 (0.002)
			0.2	0.19 (0.001)	0.59 (0.009)		0.66 (0.002)	0.58 (0.003)
								
			0.8	1.53 (0.010)	1.53 (0.006)		0.61 (0.003)	0.60 (0.002)
	A	1.63	0.5	1.32 (0.007)	1.34 (0.010)	0.60	0.63 (0.003)	0.60 (0.002)
			0.2	0.92 (0.010)	0.95 (0.008)		0.67 (0.003)	0.60 (0.003)
0.7								
			0.8	0.36 (0.002)	0.36 (0.001)		0.61 (0.003)	0.60 (0.002)
	B	0.39	0.5	0.31 (0.002)	0.32 (0.001)	0.60	0.63 (0.003)	0.60 (0.002)
			0.2	0.22 (0.002)	0.23 (0.002)		0.67 (0.003)	0.60 (0.003)
								
			0.8	0.56 (0.003)	0.63 (0.002)		0.61 (0.003)	0.60 (0.002)
	C	0.63	0.5	0.42 (0.003)	0.63 (0.003)	0.60	0.63 (0.003)	0.60 (0.002)
			0.2	0.21 (0.002)	0.63 (0.009)		0.67 (0.003)	0.60 (0.003)
								
			0.8	0.15 (0.003)	0.17 (0.003)		0.17 (0.003)	0.17 (0.003)
	cont	0.17	0.5	0.11 (0.003)	0.16 (0.004)	0.17	0.18 (0.003)	0.17 (0.003)
			0.2	0.05 (0.002)	0.17 (0.006)		0.19 (0.003)	0.17 (0.003)
								
			0.8	0.44 (0.010)	0.45 (0.010)		0.18 (0.003)	0.17 (0.003)
	A	0.47	0.5	0.38 (0.010)	0.38 (0.009)	0.17	0.18 (0.003)	0.17 (0.003)
			0.2	0.27 (0.010)	0.29 (0.010)		0.19 (0.003)	0.17 (0.003)
0.2								
			0.8	0.11 (0.002)	0.11 (0.002)		0.18 (0.003)	0.17 (0.003)
	B	0.11	0.5	0.09 (0.002)	0.09 (0.002)	0.17	0.18 (0.003)	0.17 (0.003)
			0.2	0.06 (0.002)	0.07 (0.002)		0.19 (0.003)	0.17 (0.003)
								
			0.8	0.16 (0.003)	0.18 (0.004)		0.18 (0.003)	0.17 (0.003)
	C	0.18	0.5	0.12 (0.003)	0.18 (0.004)	0.17	0.18 (0.003)	0.17 (0.003)
			0.2	0.06 (0.002)	0.18 (0.007)		0.19 (0.003)	0.17 (0.003)

Due to the introduction of *Z*, the true effects that we are trying to estimate are somewhat smaller than when *X *is the only independent variable in the models. Nevertheless, we see that when the correlation between *X *and *Z *is small (Table 3), the pattern from Table 2 is repeated, in that the naive and *RC *corrected estimates of the effects of *X *are very similar for models A and B, while for continuous exposure and for model C, *RC *is much better. In fact, the attenuation factors are quite similar to the ones obtained in Table 2 (for constant *k*).

Regarding the effects estimates for the covariate *Z*, we see that both methods are quite good, though while the *RC *approach gives unbiased estimates, the naive approach tends to overestimate as the measurement error increases. This is a well-known effect for covariates positively correlated to error-prone explanatory variables.

When the correlation between *X *and *Z *is stronger (Table 4), the differences between the naive and the *RC *corrected estimates increase, especially when the measurement error is large. Actually, the attenuation factors for the *RC *approach are about the same as in Table 3 for models A and B. Meanwhile, the naive estimates are attenuated by a factor 0.4 in the worst cases (*λ *= 0.2). So, the high correlation leads to more bias in the naive effect estimates, but it also means that the covariate *Z *contains much information about the true exposure *X*, enabling the *RC *approach to counteract parts of the bias.

**Table 4:** Results from simulations including a covariate *Z*, strongly correlated to *X*. Naive and regression calibrated effect estimates in linear regression with error-prone exposure *X *and a covariate *Z *strongly correlated to *X *(*ρ*_*XZ *_= 0.7), analysing (A) dummy variables, comparing 5th vs. 1st quintile, (B) quintile numbers, and (C) median values within quintile groups. Results from analysis with continuous exposure is included for comparison. We have *X*, *Z *and *Y *~ *N *(0,1) and the error *U *~ *N *(0, ), where  is chosen such that the reliability ratio λ is either 0.8, 0.5 or 0.2. The results are obtained via simulation, where the correlations *ρ*_*XY *_= *ρ*_*ZY *_are set to either 0.7 or 0.2. The true effects of *X *and *Z *are indicated for the various models. All individuals are measured twice. Standard errors for the corrected cases are bootstrapped.

*ρ*_*XY *_= *ρ*_*ZY *_	model	true *coef *(*X*)	*λ*	naive *coef *(*X*) (*SE*)	*RC coef *(*X*) (*SE*)	true *coef *(*Z*)	naive *coef *(*Z*) (*SE*)	*RC coe *(*Z*) (*SE*)
			0.8	0.33 (0.003)	0.41 (0.003)		0.47 (0.003)	0.41 (0.003)
	cont	0.41	0.5	0.21 (0.002)	0.40 (0.005)	0.41	0.55 (0.003)	0.41 (0.004)
			0.2	0.09 (0.001)	0.42 (0.013)		0.64 (0.002)	0.40 (0.009)
								
			0.8	0.91 (0.013)	0.97 (0.010)		0.51 (0.004)	0.47 (0.003)
	A	1.05	0.5	0.68 (0.012)	0.84 (0.010)	0.47	0.58 (0.004)	0.49 (0.003)
			0.2	0.40 (0.011)	0.59 (0.013)		0.65 (0.003)	0.53 (0.005)
0.7								
			0.8	0.21 (0.003)	0.23 (0.002)		0.51 (0.004)	0.47 (0.003)
	B	0.25	0.5	0.16 (0.003)	0.20 (0.002)	0.47	0.58 (0.004)	0.49 (0.003)
			0.2	0.09 (0.002)	0.14 (0.003)		0.65 (0.003)	0.53 (0.004)
								
			0.8	0.33 (0.004)	0.40 (0.004)		0.51 (0.004)	0.47 (0.003)
	C	0.41	0.5	0.21 (0.003)	0.37 (0.004)	0.47	0.58 (0.004)	0.49 (0.003)
			0.2	0.09 (0.002)	0.30 (0.007)		0.65 (0.003)	0.53 (0.004)
								
			0.8	0.09 (0.004)	0.12 (0.005)		0.13 (0.004)	0.11 (0.004)
	cont	0.12	0.5	0.06 (0.003)	0.11 (0.006)	0.12	0.16 (0.004)	0.12 (0.005)
			0.2	0.02 (0.002)	0.11 (0.010)		0.19 (0.003)	0.13 (0.008)
								
			0.8	0.26 (0.012)	0.27 (0.012)		0.14 (0.004)	0.13 (0.004)
	A	0.30	0.5	0.20 (0.012)	0.23 (0.014)	0.13	0.17 (0.004)	0.14 (0.005)
			0.2	0.12 (0.011)	0.16 (0.019)		0.19 (0.003)	0.16 (0.006)
0.2								
			0.8	0.06 (0.003)	0.06 (0.003)		0.14 (0.004)	0.13 (0.004)
	B	0.07	0.5	0.05 (0.003)	0.05 (0.003)	0.13	0.17 (0.004)	0.15 (0.005)
			0.2	0.03 (0.002)	0.04 (0.004)		0.19 (0.003)	0.16 (0.006)
								
			0.8	0.09 (0.004)	0.11 (0.005)		0.14 (0.004)	0.13 (0.004)
	C	0.11	0.5	0.06 (0.004)	0.10 (0.006)	0.13	0.17 (0.004)	0.14 (0.005)
			0.2	0.02 (0.002)	0.08 (0.009)		0.19 (0.003)	0.16 (0.006)

Furthermore, while for the continuous case the regression calibration approach still manages to produce unbiased estimates, we see that for model C there are some deviations for large measurement errors. We also see that the tendency of the naive approach to overestimate the effects of *Z*, as observed in Table 3, is continued here, and now the *RC *estimates are also affected.

### Example

To illustrate our results, we use data on non supplemental folate intake, total energy intake and self-reported depression from the Norwegian Women and Cancer (NOWAC) cohort study started in 1991 [22]. The data were collected by food frequency questionnaires (FFQs), and we analyze a sub replication study in which a sample of the cohort were measured a second time. The replicated subsample consists of 898 individuals with no missing data. Hence, we have *W*_*ij *_= estimated folate intake through food (in *μ*g/MJ) for individual *i *in FFQ *j*, and *Y*_*i *_= self-reported depression (yes/no) for individual *i*, where *i *= 1, ..., 898, *j *=1, 2. The prevalence of depression in the sample was 19.7%.

The folate intake, adjusted for total energy intake, was related to self-reported depression using logistic regression modelling. Using the continuous exposure, the naive odds ratio (*OR*) was estimated as 0.70 (*SE *= 0.13) for each 10 *μ*g/MJ increase in folate intake, while the regression calibration approach gave  = 0.62 (bootstrapped *SE *= 0.16). Looking at the effect of going from the first to the last quintile (model A), we found  = 0.57, with standard errors 0.15, for both approaches. The simple trend (model B) was estimated to 0.87 (SEs 0.05) for both approaches. Applying the median values in model C, the naive effect estimate was  = 0.61 (*SE *= 0.13) for each 10 *μ*g/MJ increase in folate intake, while the corrected estimate was 0.52 (*SE *= 0.15).

Clearly, all of these results are quite unstable. However, we notice that in situations where the original scale is incorporated, the regression calibration approach gives stronger effect estimates than the naive approach. In contrast, when the analysis is performed on the quintile scale, the two approaches give similar results.

The 898 individuals included in the replication study were sampled from a larger group (*n *= 19740 with no missing data) with single measurements of folate intake. Including the total group in the analysis, we got the following results: Using the continuous exposure, the naive odds ratio was 0.84 (SE = 0.03) for each 10 *μ*g/MJ increase in folate intake, while the regression calibration approach gave  = 0.75 (SE = 0.05). Under model A, we found  = 0.71 (SE = 0.04) for both approaches, and the simple trend (model B) was estimated to 0.92 with standard error 0.01, again for both approaches. Applying the median values in model C, the naive effect estimate was  = 0.78 (*SE *= 0.03) for each 10 *μ*g/MJ increase in folate intake, while the corrected estimate was 0.67 (*SE *= 0.05).

Although we now have varying numbers of replications, the two approaches still give the same results for models A and B, probably because a total of two measurements on just 4.5% of the individuals is not enough to introduce the confusion effect mentioned previously. In total, 98.7% of the individuals were classified equally with the two approaches, and none differed by more than 1 category. The overall findings regarding the comparison naive vs. *RC *approach are unchanged.

## Discussion

We find in this paper that the excellent performance of the regression calibration method for dealing with measurement error on continuous exposures in regression analysis, is diminished when the exposure is categorized before effect estimates are obtained. As shown, one needs to relate back to the original scale for the approach to be valuable.

In particular, we find that the effect estimates using *RC *are comparative to those obtained by a naive approach of not correcting for measurement error, when the exposure is analysed on a categorical scale. In some cases they are analytically equal. The main reason for the poor results is that categorizing using the corrected exposure still retains misclassification, which is similar to the misclassification obtained with the naive approach, and this misclassification induces bias in the effect estimates. When using the median measured value of each exposure group as explanatory variable, regression calibration works by decreasing the spread in the exposure distribution, thus resulting in larger effect estimates.

For regression analysis including a covariate measured without error, we find some differences between the naive and the *RC *approach, especially when the correlation between the exposure and the covariate is strong. However, none of the approaches are particularly good.

Since the reason for the poor results is to be found in the treatment of the explanatory variable, our general findings are most certainly not exclusive to any regression model, but can be extended to concern other regression models.

In diagnostic tests, for example, it is quite common to categorize according to a fixed cut-off level, where an extreme value is diagnosed as a case. Furthermore, in epidemiologic studies, one can also relate to fixed exposure groups/exposure groups that are defined independently from the observed data, classifying for example smoking into {0}, {1 – 10}, {11 – 20} and {> 20} cigarettes per day, or body mass index (BMI) into underweight (< 18.5), normal weight (18.5 to 24.9), overweight (25 to 29.9), and obese (≥ 30). A small simulation study was conducted to explore whether the current results sustain when such fixed cut-points are applied, and it seems *RC *now gains a small advantage compared to the naive approach. Also, the more extreme the cut-point, the larger the difference between the two approaches. This situation corresponds to the one where the true percentiles are known, though the interpretation of the results is somewhat different.

We have focused on a situation with replicates. However, as outlined in the Introduction, other sources of information regarding the measurement error could be either internal or external validation studies or instrumental variables. The approach studied in this paper would still amount to fitting a regression model for the true given the measured exposure, and including the predicted exposure from this model in the main analysis. Furthermore, the percentiles would be predicted by the same model, so naive and corrected categorized exposure are the same in these situations as well.

In some cases it might not be appropriate to use the original scale in the analysis, the researcher might specifically wish to relate to the categorical variables. In our view, there are two possible approaches to obtain efficient effect estimates in these cases. Either a) some information is needed about misclassification probabilities or b) a better way is needed to categorize from the original continuous measurements.

We cannot achieve a) using just replicate measures (without further assumptions on the distribution of *X*) but could if we had validation data. For example, Rosner [16] suggested to simply treat these situations as misclassification problems, using ordinal regression procedures with validation data. A similar approach involving latent class modeling of replicated data has been proposed [23]. Recently, Küchenhoff *et al. *[24] developed the MC-SIMEX methodology, to deal with situations with misclassification in categorical exposure and/or response, however the procedure requires either knowledge or an estimate of the misclassification matrix. A Bayesian approach to misclassification problems has been suggested [25], which might be taken a step further in our setting.

To achieve b) one can try to estimate the underlying distribution of *X*, and its percentiles in a nonparametric way using the replicate measures. There has been extensive work on estimating the distribution of *X *(see [26] and references therein, and a new idea recently proposed by Freedman *et al. *[27]) but the ability of these techniques to accurately estimate percentiles has not been fully explored. Work is underway to explore the use of these techniques in the current problem.

Instead of going via the expected values of the continuous exposure, we could find directly the expected categorical exposure. We expect that analysis with expected conditional probabilities (given the observed exposure) of the categories will give better results than the analysis with dummy variables. The latter amounts to adjusting the probability of the most probable category to 1 and all the other probabilities to 0, thereby disregarding the information that lies in the uncertainty of the categorization.

Future work should aim to develop suitable and functional correction procedures in analyses where the exposure variable is categorized according to percentiles, and investigations should be carried out in order to decide which method is the best or most suitable for recommendations to include in routine analysis.

## Competing interests

The author(s) declare that they have no competing interests.

## Authors' contributions

ID was responsible for most of the study design, analysis and writing. JPB, PL and MT helped with the conceptualization and writing of the article, AH did the data preparation.

## References

[B1] Fuller W (1987). Measurement Error Models Wiley Series in Probability and Mathematical Statistics: Probability and Mathematical Statistics.

[B2] Carroll R, Ruppert D, Stefanski L (1995). Measurement Error in Nonlinear Models.

[B3] Rosner B, Willett W, Spiegelman D (1989). Correction of Logistic Regression Relative Risk Estimates and Confidence Intervals for Systematic Within-Person Measurement Error. Statistics in Medicine.

[B4] Rosner B, Spiegelman D, Willett W (1990). Correction of Logistic Regression Relative Risk Estimates and Confidence Intervals for Measurement Error: The Case of Multiple Covariates Measured with Error. American Journal of Epidemiology.

[B5] Carroll R, Stefanski L (1990). Approximate Quasilikelihood Estimation in Models with Surrogate Predictors. Journal of the American Statistical Association.

[B6] Hardin JW, Schmiediche H, Carroll RJ (2003). The Regression-Calibration Method for Fitting Generalized Linear Models with Additive Measurement Error. The Stata Journal.

[B7] SAS macros for regression calibration for main study/validation study design. http://www.hsph.harvard.edu/faculty/spiegelman/blinplus.html.

[B8] SAS macros for regression calibration for main study/reliability study design. http://www.hsph.harvard.edu/faculty/spiegelman/relibpls8.html.

[B9] Weuve J, Kang J, Manson J, Breteler M, Ware J, Grodstein F (2004). Physical Activity, Including Walking, and Cognitive Function in Older Women. JAMA.

[B10] Hoffmann K, Zyriax B, Boeing H, Windier E (2004). A Dietary Pattern Derived to Explain Biomarker Variation is Strongly Associated with the Risk of Coronary Artery Disease. American Journal of Clinical Nutrition.

[B11] Lahmann P, Hoffmann K, Allen N, Gils CV, Khaw K, Tehard B, Berrino F, Tjønneland A, Bigaard J, Olsen A, Overvad K, Clavel-Chapelon F, Nagel G, Boeing H, Trichopoulos D, Economou G, Bellos G, Palli D, Tumino R, Panico S, Sacerdote C, Krogh V, Peeters P, de Mesquita HB, Lund E, Ardanaz E, Amiano P, Pera G, Quirós J, Martínez C, Tormo M, Wirfält E, Berglund G, Hallmans G, Key T, Reeves G, Bingham S, Norat T, Biessy C, Kaaks R, Riboli E (2004). Body Size and Breast Cancer Risk: Findings from the European Prospective Investigation Into Cancer and Nutrition (EPIC). Int J Cancer.

[B12] Smith G, Wood A, Pell J, White I, Crossley J, Dobbie R (2004). Second-Trimester Maternal Serum Levels of Alpha-Fetoprotein and the Subsequent Risk of Sudden Infant Death Syndrome. The New England Journal of Medicine.

[B13] Schaumberg D, Liu S, Seddon J, Willett W, Hankinson S (2004). Dietary Glycemic Load and Risk of Age-Related Cataract. American Journal of Clinical Nutrition.

[B14] Shai I, Rimm E, Hankinson S, Cannuscio C, Curhan G, Manson J, Rifai N, Stampfer M, Ma J (2005). Lipoprotein (a) and Coronary Heart Disease among Women: Beyond a Cholesterol Carrier?. European Heart Journal.

[B15] Al-Zahrani M (2006). Increased Intake of Dairy Products Is Related to Lower Periodontitis Prevalence. Journal of Periodontology.

[B16] Rosner BA (1996). Measurement Error Models for Ordinal Exposure Variables Measured with Error. Statistics in Medicine.

[B17] Prentice R (1982). Covariate Measurement Errors and Parameter Estimation in a Failure Time Regression Model. Biometrika.

[B18] Armstrong B (1985). Measurement Error in Generalized Linear Models. Communications in Statistics Series B.

[B19] Gleser L, Brown P, Fuller W (1990). Improvements of the Naive Approach to Estimation in Nonlinear Errors-in-Variables Regression Models. Statistical Analysis of Measurement Error Models and Application.

[B20] Efron B, Tibshirani R (1993). An Introduction to the Bootstrap.

[B21] The R Project for Statistical Computing. http://www.r-project.org.

[B22] Lund E, Kumle M, Braaten T, Hjartåker A, Bakken K, Eggen E, Gram I (2003). External Validity in a Population-based National Prospective Study--the Norwegian Women and Cancer Study (NOWAC). Cancer Causes Control.

[B23] Albert P, McShane L, Shih J, Network TUNCIBTM (2001). Latent Class Modeling Approaches for Assessing Diagnostic Error without a Gold Standard: With Applications to p53 Immunohistochemical Assays in Bladder Tumors. Biometrics.

[B24] Küchenhoff H, Mwalili S, Lesaffre E (2006). A General Method for Dealing with Misclassification in Regression: The Misclassification SIMEX. Biometrics.

[B25] Gustafson P (2004). Measurement Error and Misclassification in Statistics and Epidemiology : Impacts and Bayesian Adjustments.

[B26] Böhning D (1995). A Review of Reliable Maximum Likelihood Algorithms for Semiparametric Mixture Models. Journal of Statistical Planning and Inference.

[B27] Freedman L, Fainberg V, Kipnis V, Midthune D, Carroll R (2004). A New Method for Dealing with Measurement Error in Explanatory Variables of Regression Models. Biometrics.

